# TinyML-Based In-Pipe Feature Detection for Miniature Robots

**DOI:** 10.3390/s25061782

**Published:** 2025-03-13

**Authors:** Manman Yang, Andrew Blight, Hitesh Bhardwaj, Nabil Shaukat, Linyan Han, Robert Richardson, Andrew Pickering, George Jackson-Mills, Andrew Barber

**Affiliations:** School of Mechanical Engineering, University of Leeds, Leeds LS2 9JT, UK; a.j.blight@leeds.ac.uk (A.B.); h.bhardwaj@leeds.ac.uk (H.B.); n.shaukat@leeds.ac.uk (N.S.); l.y.han@leeds.ac.uk (L.H.); r.c.richardson@leeds.ac.uk (R.R.); a.d.pickering@leeds.ac.uk (A.P.); g.jackson-mills@leeds.ac.uk (G.J.-M.); a.r.barber@leeds.ac.uk (A.B.)

**Keywords:** tiny machine learning (TinyML), resource-efficient, miniature robot, in-pipe feature detection, convolutional neural network (CNN)

## Abstract

Miniature robots in small-diameter pipelines require efficient and reliable environmental perception for autonomous navigation. In this paper, a tiny machine learning (TinyML)-based resource-efficient pipe feature recognition method is proposed for miniature robots to identify key pipeline features such as elbows, joints, and turns. The method leverages a custom five-layer convolutional neural network (CNN) optimized for deployment on a robot with limited computational and memory resources. Trained on a custom dataset of 4629 images collected under diverse conditions, the model achieved an accuracy of 97.1%. With a peak RAM usage of 195.1 kB, flash usage of 427.9 kB, and an inference time of 1693 ms, the method demonstrates high computational efficiency while ensuring stable performance under challenging conditions through a sliding window smoothing strategy. These results highlight the feasibility of deploying advanced machine learning models on resource-constrained devices, providing a cost-effective solution for autonomous in-pipe exploration and inspection.

## 1. Introduction

Pipelines serve as critical infrastructure in urban and industrial environments, supporting essential industries like oil, gas, and water supply. However, as pipelines age and sustain damage, significant losses can occur, highlighting the need for regular inspections and maintenance to maintain safety and operational efficiency. Traditional inspection methods, such as manual techniques and closed-circuit television (CCTV) systems, face challenges due to the complex layouts, extensive lengths, and low-light conditions commonly found in pipelines [1]. To address these challenges, autonomous in-pipe robots have emerged as a promising solution, enabling access to pipelines with intricate configurations and those buried underground, facilitating effective inspection and detection tasks [2].

Pipelines are generally categorized by diameter: small (75–200 mm), medium (250–400 mm), and large (450–900 mm) [3]. Sewer pipelines, typically measuring between 0.1 and 1 meter in diameter, pose substantial challenges for manual inspection, as their dimensions prevent direct human access [4]. Miniature in-pipe robots have gained attention as a practical solution for navigating and inspecting these small-diameter pipelines [5]. A key challenge for these robots is feature detection, which directly influences their ability to navigate, inspect, and operate autonomously in uncertain pipe environments. Feature detection methods for in-pipe robots are typically divided into range-based and vision-based approaches [2].

Range-based methods rely on sensors to measure distances, detecting features such as elbows, joints, branches, or constrictions. For example, Nguyen et al. [3] developed a miniature robot equipped with three time-of-flight (ToF) range sensors to operate in 75 mm diameter pipelines. The robot detected features by monitoring variations in distance measurements across the front, left, and right directions. Significant changes in sensor readings indicated the presence of features, enabling autonomous navigation and feature recognition. However, range-based sensors have inherent challenges, particularly instability caused by gear backlash. This instability becomes more pronounced when the misalignment of the wheel-legs induces vertical oscillations in the robot’s body, resulting in inconsistent measurements from the range sensors, thereby compromising the accuracy and reliability of the robot. Kim et al. [6] introduced a weaving laser vision system (LVS) for autonomous mobile robots navigating pipelines, focusing on detecting T-junctions and elbow pipes. Using a 2D laser scanner with a weaving motor, the system creates selective 3D forward maps to identify pipeline features. Partial weaving is used for T-junction detection, while full weaving detects elbow angles and curvatures, modeled as sections of a torus. Experiments validated its effectiveness in controlled environments, but limitations include assumptions of empty pipelines and sensitivity to pitch and yaw.

Vision-based methods utilize cameras and illuminators to detect pipeline features. Ahrary et al. [7] proposed a traditional computer vision-based navigation method for an autonomous sewer robot KANTARO. KANTARO is able to move in 200–300 mm diameter sewer pipes. By using stereo camera information, the approach extracts feature pixels from regions of interest (ROI) in stereo images and detects local features like manholes and pipe joints through edge detection. While computationally efficient, the approach lacks robustness to lighting changes and noise. Edwards et al. [8] introduced a robust vision-based approach for pipe robots detecting features in feature-sparse sewer pipes, employing an electro-optical forward-facing camera. Pipe joints are detected using a bag-of-keypoint algorithm leveraging speeded-up robust features (SURFs), while manholes are identified with a linear support vector machine (SVM) classifier. The detection algorithms underwent unsupervised offline training and were tested on 860 frames from three real-world pipes with diameters of 600, 300, and 150 mm. The proposed detection method achieved 85% accuracy in joint detection, far outperforming the Hough Transform’s 25% accuracy. The accuracy of detecting a manhole is 98.5%. The inclusion of a windowing mechanism further enhanced detection robustness, ensuring improved reliability in challenging conditions.

Some researchers combined both range-based and vision-based methods into a single in-pipe robot. Li et al. [9] introduced a real-time topological localization and mapping system for an autonomous in-pipe miniature robot that was developed from Nguyen et al. [3]. It combines low-power distance sensors for navigation with vision-based localization, activating the camera only at junctions to save energy and reduce computation. Junctions are mapped as nodes and pipeline segments as edges, creating a compact, dynamically updated topological map. Using normalized cross-correlation for image matching, the system effectively identifies and maps junctions in a simulated pipe network, even under imperfect conditions. While the method excels in simulation, challenges like sensor limitations and environmental factors in real-world deployment remain. Lee et al. [10,11,12] have performed a series of experiments using range and visual sensors for pipeline feature detection respectively. Additionally, they combined both range-based and vision-based methods into a single in-pipe robot MRINSPECT-V to detect natural gas pipeline (203 mm in diameter) features under different scenarios. The vision-based systems were equipped with 64 high-brightness LEDs and a camera that used distinct shadow patterns created by the robot’s illuminator to identify features, leveraging traditional image-processing techniques such as binarization, morphological operations, and Hu invariant moments for feature extraction. The range-based method utilizes a rotating line-laser beam to detect geometric features, offering enhanced robustness in contaminated environments. The experimental results demonstrate that, while the vision-based method is computationally efficient, it is sensitive to noise and contamination, whereas the range-based method is more resilient but computationally intensive, making both approaches complementary for different pipeline conditions [10,11,12].

Vision-based methods offer notable advantages over range-based approaches by capturing detailed contextual information, including textures, colors, and patterns, making them well-suited for complex feature analysis, object recognition, and machine learning applications. These methods are versatile, cost-effective, and capable of detecting objects over longer distances under favorable lighting conditions. However, most current vision-based pipeline feature detection methods rely on traditional techniques, such as edge detection and geometric fitting. Although these approaches are computationally efficient and perform well in controlled settings, their reliance on handcrafted features such as manually defined edge patterns or geometric rules limits their robustness in scenarios with unpredictable noise, dynamic lighting, or intricate textures, commonly encountered in real-world environments.

In contrast, machine learning-based methods, particularly deep learning, automatically learn feature representations from data. As a class of deep learning model, CNN uses convolutional layers to efficiently handle high-dimensional image data without losing critical features, and it has proved its excellent ability in computer vision over the years. This enables higher accuracy, improved robustness to environmental variations, and adaptability to diverse pipeline conditions [13]. However, deep learning models typically require significant computational and storage resources for training and deployment. Addressing this challenge, TinyML has emerged as a solution, compressing deep learning models to enable deployment on resource-constrained devices, such as internet of things (IoT) devices and microcontrollers. TinyML allows machine learning applications to operate within a few hundred kilobytes of memory and milliwatt-level power consumption [14,15,16].

Recent studies demonstrated the potential of TinyML for object classification or pipeline feature detection in resource-constrained environments. Pleterski et al. [17] applied TinyML into ultra-low resolution ToF sensors for a miniature mobile robot’s object classification, achieving 91.8% detection accuracy with high inference speed. Avellaneda et al. [18] designed a TinyML assisted IoT method for indoor asset tracking and identification. The method can achieve a classification accuracy of 88%. Wang et al. [19] demonstrated high accuracy, highlighting the potential of deep learning for pipeline feature detection in complex environments (120 mm in inner diameter). The authors utilized ResNet18 to classify four types of features: straight, curved, and T-shaped pipes. A dataset of 908 images captured under diverse conditions was used to train the model, achieving high accuracy and addressing the limitations of traditional methods. However, the study did not address the computational and memory constraints of miniature pipeline robots. Furthermore, the ResNet18 model was not deployed on the actual π-II robot, leaving critical challenges, such as onboard processing and real-world testing, unresolved.

The deployment of miniature mobile robots in pipeline environments remains a relatively unexplored area. Leveraging TinyML for feature detection in such constrained settings introduces a range of challenges. A primary limitation is the scarcity of comprehensive datasets that adequately represent the diversity of pipeline features across varying scenarios. Additionally, existing lightweight neural network architectures, such as the MobileNet series [20,21,22], may not be optimized for the unique requirements of this application domain. Beyond accuracy, critical considerations such as inference time and computational efficiency are paramount when selecting models suitable for deployment on resource-constrained, cost-sensitive miniature robots.

This paper focuses on pipeline feature detection, specifically tailored for a miniature mobile robot operating in constrained environments. A specialized pipeline feature dataset was first collected to support model training and evaluation under diverse scenarios. To identify the most suitable learning-based approach, different CNN architectures were compared based on factors such as accuracy and resource constraints. The main contributions of this paper are fourfold: (1) Custom dataset development: A dedicated pipeline feature dataset, capturing various scenarios, is collected to support training and evaluation under diverse conditions. (2) Optimized CNN selection: A thorough comparison of CNN architectures is conducted, resulting in the selection of a custom CNN model optimized for resource-constrained environments by balancing key metrics such as inference time and accuracy. (3) High accuracy detection: Accurate detection (>90%) by a miniature mobile robot in small pipes is achieved using a camera and CNNs implemented on a low-end microcontroller (ESP32 with a 240 MHz CPU and 520 kB RAM). (4) Robust and real-time results: A sliding window data smoothing method is combined with the selected CNN model to enhance robustness and enable real-time performance. This method addresses the unique challenges of pipeline exploration by combining computational efficiency, robust detection, and adaptability to diverse environments, demonstrating its potential for real-world applications in autonomous robotic navigation.

The rest of the paper is structured as follows. Section 2 outlines the methodology employed in this work. Section 3 presents the evaluation results of the CNN model and the data smoothing techniques. Section 4 discusses the limitations of this study and suggests directions for future research. Finally, Section 5 concludes the paper with a summary of the findings.

## 2. Materials and Methods

### 2.1. Dataset Collection

To enable effective navigation of unknown pipelines and ensure critical exploration opportunities are not missed, miniature robots require the ability to detect key pipeline features such as elbows, joints, and left turns. To support this, a custom dataset was specifically developed for training TinyML models.

**Figure 1:**
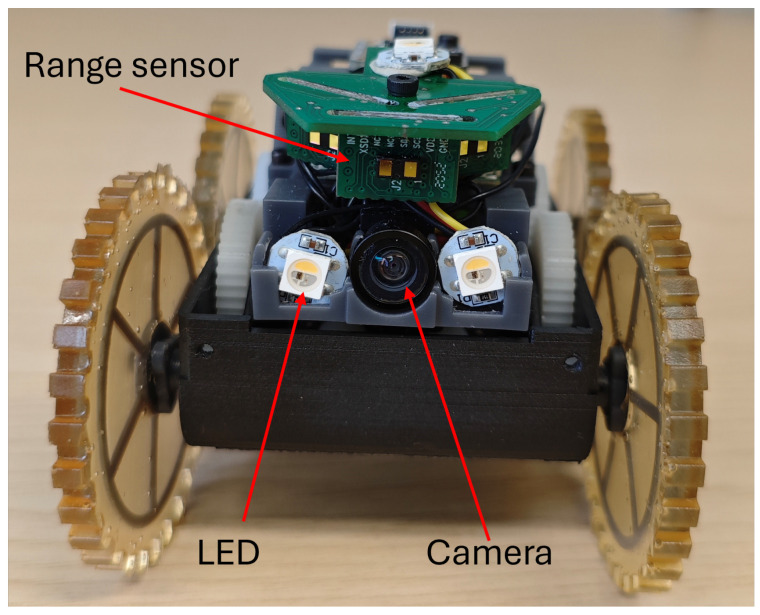
Miniature pipeline robot platform.

This study employs a miniature in-pipe mobile robot named Joey, which originated from the Pipebots [23] project. The Joey robot was designed to be carried and deployed by a larger robot for inspecting smaller sewer pipes. The robot used in this work builds upon the original Joey design, incorporating an upgraded frame and motor while retaining the same sensor setup. Joey is equipped with two LED lights and an OV2640 camera mounted at the front, as illustrated in Figure 1.

**Figure 2:**
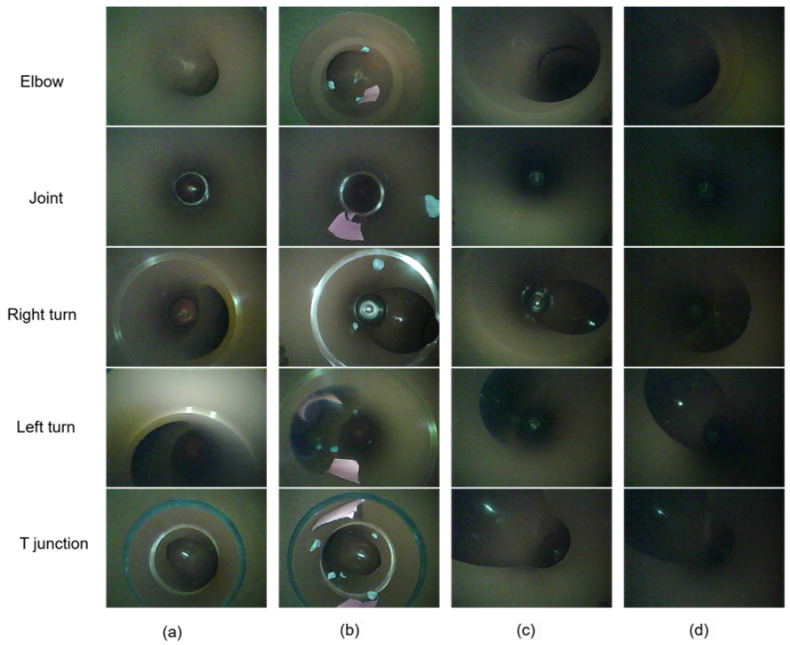
Images of pipe features captured under different conditions: (**a**) 110 mm pipe, (**b**) 110 mm pipe with interference, (**c**) 160 mm pipe, (**d**) 160 mm pipe with low-light source.

The dataset comprises 4629 unique images, collected under four distinct conditions to enhance the robustness and adaptability of the TinyML algorithm. These conditions include two pipe diameters (110 mm and 160 mm), low-light environments, and simulated interference. Interference within the pipeline was emulated by placing shredded paper and plasticine inside the pipe, mimicking realistic operational scenarios. Low-light conditions were achieved by deactivating one of the LED lights during image acquisition. Figure 2 illustrates examples of images captured under these various conditions. This diverse dataset aims to improve the algorithm’s performance across varying real-world pipeline environments.

**Figure 3:**
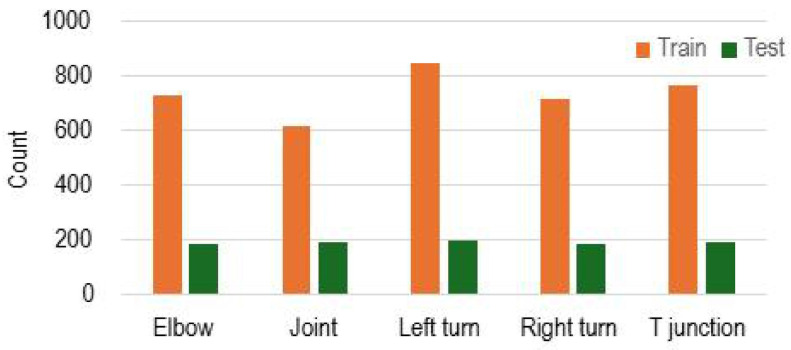
Distribution of the pipeline feature dataset.

The distribution of the pipeline feature dataset is shown in Figure 3. The dataset consists of five classes, which are elbow, joint, left turn, right turn, and T-junction. Images were labeled manually for pipeline feature recognition. To evaluate the machine learning model and escape over-fitting of the model, the dataset is approximately separated into 80% training images and 20% test images. Slight variations exist to ensure even test samples and to maintain integer sample counts during partitioning. These adjustments help provide a fair and reliable performance evaluation across all classes.

### 2.2. Training

To identify an optimal CNN architecture for pipeline feature detection, various neural network models were evaluated. These models were classified into two categories: training from scratch and transfer learning.

Training from scratch involves building a model entirely from the ground up, with randomly initialized weights. The model learns feature representations directly from the provided dataset without utilizing prior knowledge. This traditional approach requires significant computational resources and a sufficiently large dataset to achieve high performance. In contrast, transfer learning leverages knowledge from a pre-trained model, typically trained on a large, generic dataset, and applies it to a new, task-specific dataset. This method mitigates challenges associated with limited data availability and significantly reduces training time and computational cost.

The evaluation was conducted using Edge Impulse [24], a platform that provides pre-trained MobileNetV2 models [21] with various depth multipliers. The dataset described in Section 2.1 was imported into the platform for training. To ensure efficient deployment on low-resource devices, 8-bit quantization was applied to the models. Quantization is a widely adopted model compression technique that reduces computational and storage requirements. Random seeds were set during training to control the inherent randomness across different stages of the training and evaluation processes, ensuring consistent and reproducible experimental results [25]. Fixing random seeds is a commonly adopted practice in experimental research [26]. The performance metrics, including accuracy, loss, inference time, peak RAM usage, and flash usage, were systematically compared, as presented in Table 1. Unless specified otherwise, the default input image size was 96×96.

A convolutional layer is a key part of CNNs that extracts features from data, like edges or patterns in images. It applies small filters across the input, detecting local details while sharing parameters to reduce complexity. This makes CNNs efficient and effective for image analysis. Models trained from scratch included architectures with varying depths (2, 3, 4, 5, and 6 convolutional layers (Conv)), models with larger input sizes (160×160), architectures incorporating DepthwiseConv2D and PointwiseConv2D (Conv+DW+PW+Conv), and models enhanced with attention mechanisms such as squeeze-and-excitation (SE) [27] and efficient channel attention (ECA) [28] (e.g., 3 Conv+SE and 3 Conv+ECA). DepthwiseConv2D (DW) combined with PointwiseConv2D (PW) forms a depthwise separable convolution, an efficient alternative to standard convolution that is frequently utilized in lightweight architectures like MobileNet [22,29].

Transfer learning utilized MobileNetV1 [20] and MobileNetV2 [21] architectures with different depth multipliers, capitalizing on their lightweight design and proven efficiency in resource-constrained environments.

The experimental results revealed that models trained from scratch generally achieved higher accuracy with deeper architectures. For example, the 5 Conv attained the highest accuracy (0.971) but at the expense of higher resource consumption, including flash usage (427.9 kB) and inference time (1693 ms). Interestingly, increasing the depth further (6 Conv) led to higher resource demands but lower accuracy (0.967). On the other hand, simpler architectures like the 2 Conv demonstrated greater resource efficiency but achieved a lower accuracy of 0.849.

Input size also played a significant role in resource utilization: models with larger input dimensions (160×160) required more storage and longer inference times compared to those with 96×96, making them less practical for low-end microcontroller deployment. Additionally, modifications such as attention mechanisms (SE and ECA) and depthwise separable convolutions (DW+PW), contrary to expectations, failed to improve performance. Instead, these adjustments resulted in reduced accuracy and increased inference times, highlighting the trade-offs involved in optimizing models for resource-constrained environments.

Transfer learning models, such as MobileNetV2, strike a balance between efficiency and performance. For example, MobileNetV2 with a width multiplier of 0.35 achieves an accuracy of 0.90 while maintaining moderate resource usage compared to other MobileNet variants. However, transfer learning models with significantly lower width multipliers, such as MobileNetV1 with a width multiplier of 0.1, exhibit substantial resource savings at the cost of reduced accuracy. In comparison, CNN models trained from scratch generally outperform MobileNet-based transfer learning models in terms of accuracy, albeit with higher resource requirements for deeper architectures.

The ESP32 board, with 520 kB of RAM and 4 MB of flash memory, imposes strict constraints on the selection of machine learning models for deployment. To meet these resource limitations while ensuring high performance, the 5 Conv model was selected as the final architecture for pipeline feature detection. This model strikes an effective balance between accuracy and resource efficiency, making it an optimal choice for the application.

**Figure 4:**
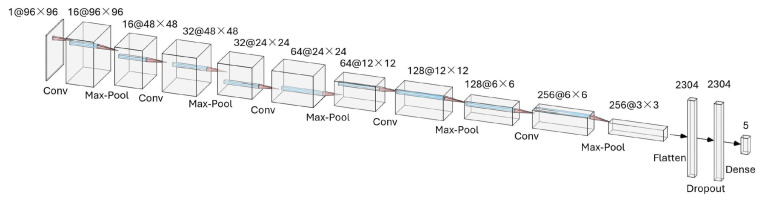
The selected CNN architecture.

The architecture of the selected model is illustrated in Figure 4. It consists of five convolutional layers, each with a kernel size of 3, followed by max pooling layers with a stride of 2. The model also includes a flatten layer to prepare the data for subsequent dense layers, a dropout layer to prevent overfitting and a final dense layer for classification. The model is trained using categorical cross-entropy loss, which is standard for multi-class classification tasks. The functionality of each layer is detailed below.

Input Layer: The input to the model is a grayscale image of size 96×96, denoted as $I\in R^{96\times 96\times 1}$. The image represents the raw pipeline scene captured by the onboard camera.

Convolutional Layers: The model employs five convolutional layers, each followed by a max-pooling layer for spatial downsampling. Each convolutional operation is defined as follows:

(1) $$O_{i,j,k}=\sigma \left(\sum_{m,n,c}I_{i+m,j+n,c}\cdot K_{m,n,c,k}+b_{k}\right),$$

where

- $O_{i,j,k}$ is the output feature map;
- $I_{i+m,j+n,c}$ is the input at location (i+m,j+n) in channel *c*;
- $K_{m,n,c,k}$ is the convolution kernel with *k* output channels;
- $b_{k}$ is the bias term for the *k*-th filter;
- σ is the activation function, in this case, ReLU:
  (2) ReLU(x)=max(0,x).

Each convolutional layer has the following configuration:

- Layer 1: 16 filters of size 3×3.
- Layer 2: 32 filters of size 3×3.
- Layer 3: 64 filters of size 3×3.
- Layer 4: 128 filters of size 3×3.
- Layer 5: 256 filters of size 3×3.

Pooling Layers: After each convolutional layer, a max-pooling operation is applied to reduce spatial dimensions. The max-pooling operation is defined as follows:

(3) $$P_{i,j,k}=\max_{\left(m,n)\in R(i,j\right)}O_{i+m,j+n,k},$$

where R(i,j) represents the pooling region. A pooling size of 2×2 with stride 2 is used.

Fully Connected Layer: The feature maps are flattened into a 1D vector and passed to a fully connected dense layer. This layer maps the features to *C* output classes using the Softmax activation function:

(4) $$y_{k}=\frac{e^{z_{k}}}{\sum_{j=1}^{C}e^{z_{j}}},$$

where $z_{k}$ is the output of the *k*-th neuron before activation.

Dropout Regularization: To prevent overfitting, a dropout layer with a rate of 0.25 is applied before the dense layer.

### 2.3. Data Smoothing

**Figure 5:**
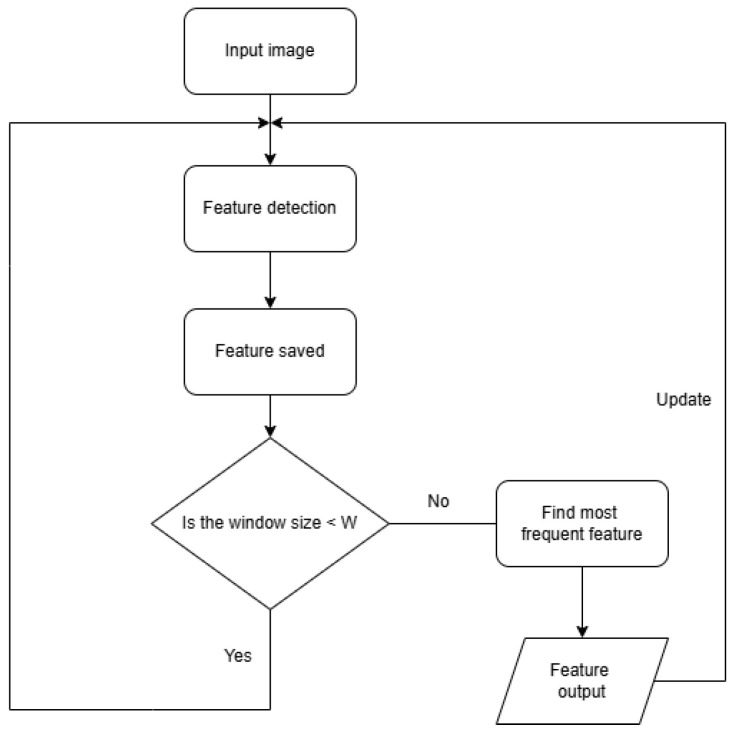
The flowchart of sliding window smoothing method.

To minimize the impact of errors from single-frame detection and obtain a more reliable and stable detection result, a sliding window smoothing strategy is used. The flowchart of the strategy is shown in Figure 5. The specific process is as follows:

Input image: The process begins with capturing an input image, which serves as the raw data for the pipeline feature detection method. This image is typically taken by the robot’s camera while it navigates the pipeline.

Feature detection: The method processes the image to identify specific pipeline features, such as elbows, right turns, or joints. The feature detection step relies on the model’s ability to classify features from the image.

Feature saved: Once a feature is detected, it is saved into a sliding window buffer. Let $F_{t}$ represent the feature detected at time *t*. A sliding window of size *W* is defined, maintaining a sequence of the last *W* detected features:

(5) $$F=\{F_{t-W+1},F_{t-W+2},\ldots ,F_{t}\}.$$

Find most frequent feature: The final detected feature $F_{\mathrm{output}}$ is determined using majority voting within the sliding window:

(6) $$F_{\mathrm{output}}=\arg\max_{f}\sum_{i=t-W+1}^{t}I\left(F_{i}=f\right),$$

where:

- $I(F_{i}=f)$ is the indicator function, defined as follows:
  (7) $$I\left(F_{i}=f\right)=\left\{\begin{array}{cc} 1, & \mathrm{if}\;F_{i}=f,\\ 0, & \mathrm{otherwise}. \end{array}\right.$$
- *f* represents a possible feature class.

Feature output: The most frequent feature from the sliding window is then outputted as the final detected feature. This ensures that the algorithm’s decision is not overly influenced by any single erroneous frame. After outputting the final feature, the algorithm goes back to capturing the next input image and repeats the process. As new features are detected, they replace the oldest feature in the sliding window, ensuring the size of the window remains constant at *W*.

**Figure 6:**
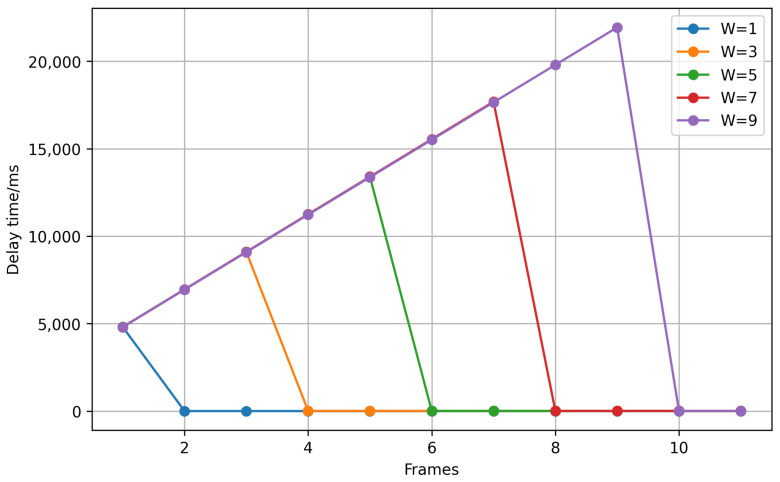
The delay time of different W values.

The delay time for different *W* values is analyzed in Figure 6. The results show that a larger *W* increases the initial delay, as more frames are needed to establish a stable prediction. However, once enough frames have been processed, the delay stabilizes since the time required to determine the most frequent feature remains nearly constant. Additionally, *W* influences the smoothness of feature transitions. When a feature change occurs, a larger *W* extends the transition period, requiring more frames and leading to a longer adjustment time.

Therefore, the choice of *W* is critical to balancing robustness and responsiveness:

- A larger *W* increases stability by smoothing transient misclassifications but introduces latency in adapting to new features.
- A smaller *W* reduces latency, enabling quicker responses to changes, but may result in less robust smoothing.

An optimal value of *W* is selected empirically to suit the pipeline detection task, ensuring reliable performance under diverse conditions.

By using this sliding window smoothing strategy, the impact of transient errors or noise in single frames can be minimized. The mechanism ensures that detections are consistent across multiple frames, providing more reliable results for navigation or decision-making in the pipeline environment.

## 3. Results

### 3.1. Model Evaluation

In this work, the test datasets consist of 958 images. To evaluate the model comprehensively, F1 score, overall accuracy, uncertainty, optimal threshold, and the receiver-operating characteristic (ROC) analysis together with the area under the curve (AUC) score are analyzed.

The ROC curve is a graphical representation of a classification model’s performance across various thresholds. It plots the true positive rate (TPR) on the y-axis against the false positive rate (FPR) on the x-axis, providing insights into the model’s ability to distinguish between classes at different decision thresholds. AUC is a scalar metric derived from the ROC curve, quantifying the model’s overall discriminative performance. The TPR and FPR are calculated using Equations (8) and (9), where TP, FP, TN, and FN denote true positives, false positives, true negatives, and false negatives, respectively.

(8) $$TPR=\frac{TP}{TP+FN}$$

(9) $$FPR=\frac{FP}{FP+FN}$$

**Figure 7:**
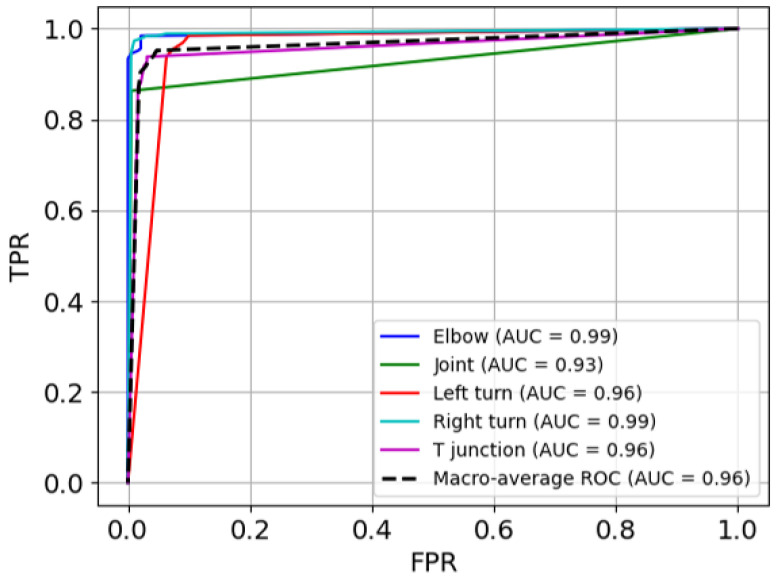
ROC curves.

The ROC curves of the model are presented in Figure 7, illustrating its overall efficacy in feature classification and its discriminative performance across all categories. The macro-average AUC is 0.96, signifying excellent and robust overall performance. Notably, the elbow and right turn categories achieve near-perfect AUC values of 0.99, demonstrating the model’s strong classification capability for these classes. The left turn and T-junction categories also perform well, with AUC values of 0.96. However, the joint category exhibits a slightly lower AUC of 0.93, indicating a relative challenge in distinguishing it from other categories.

The F1 score is a metric used to evaluate the performance of a classification model, particularly when there is an imbalance between classes. It is the harmonic mean of precision and recall, providing a single measure that balances these two aspects. The formula is shown in Equation (10).

(10) $$F1=2\times \frac{Precision\times Recall}{Precision+Recall}$$

Precision measures the proportion of correctly predicted positive instances out of all instances predicted as positive. The formula is shown in Equation (11).

(11) $$Precision=\frac{TP}{TP+FP}$$

Recall measures the proportion of correctly predicted positive instances out of all actual positive instances. The formula is shown in Equation (12).

(12) $$Recall=\frac{TP}{TP+FN}$$

**Figure 8:**
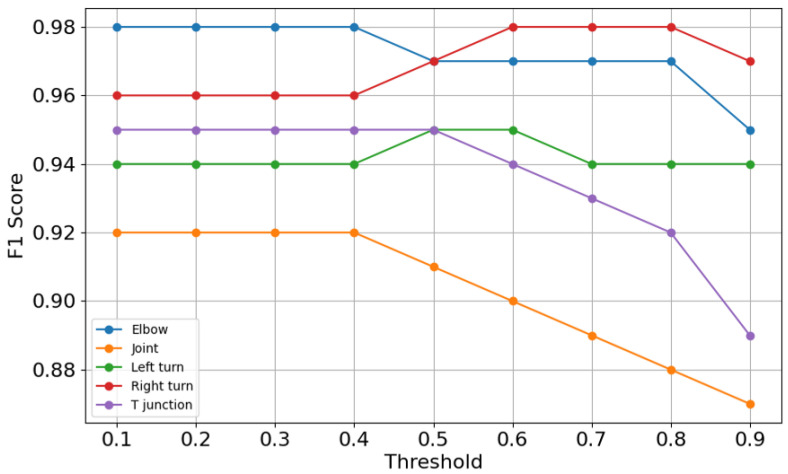
F1 score curves.

**Figure 9:**
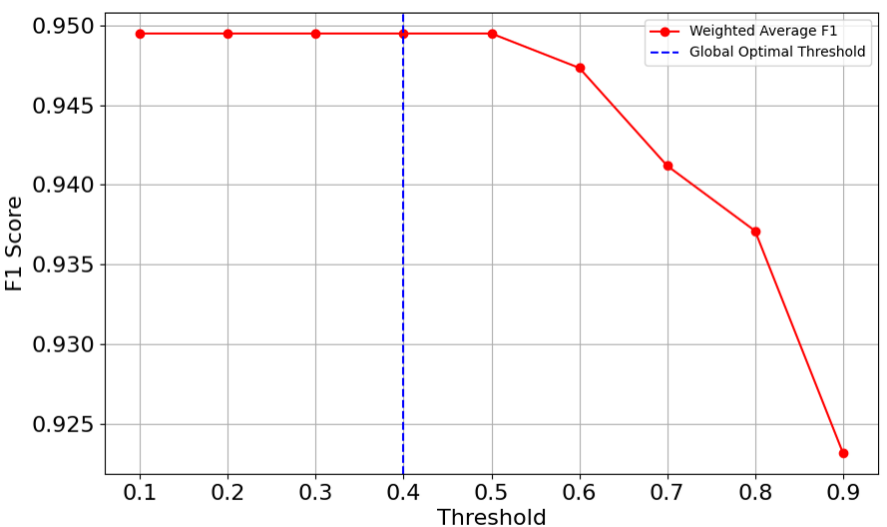
Optimal threshold.

Figure 8 and Figure 9 offer insights into each category’s F1 score and overall F1 score that evolve with varying thresholds respectively. Threshold and W serve different purposes. The threshold defines the minimum confidence required for a neural network to make a prediction. In contrast, W determines the size of the sliding window, which helps reduce misclassification effects by smoothing predictions across consecutive samples. In Figure 8, it can be seen that each category keeps stable F1 score before the threshold of 0.4. After the threshold of 0.4, each category performs differently: joint and elbow drops, while right turn and left turn have temporary rises and T-junction shows a decline after the threshold of 0.5. These findings confirm that setting a threshold too high undermines the model’s ability to classify features correctly, particularly for less robust categories like joint.

In Figure 9, it can be seen that the weighted average F1 score is consistently high at approximately 0.95 between 0.1 and 0.5, reflecting the model’s high overall performance in balancing precision and recall. As the threshold increases beyond 0.5, the weighted F1 score begins to decline, indicating that stricter thresholds disproportionately penalize weaker categories like joint and left turn. Given that the F1 score for some categories, such as joint, drops to the threshold of 0.5, 0.4 was chosen as the optimal global threshold for this model to balance precision and recall.

**Figure 10:**
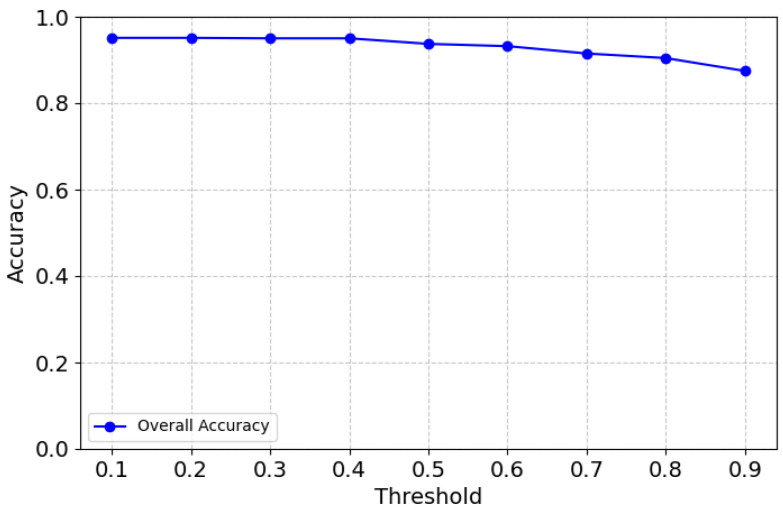
Overall accuracy.

The overall accuracy of the model is shown in Figure 10. The graph shows that the model maintains a high and stable accuracy between thresholds 0.1 and 0.4, with a slight decline beyond the threshold of 0.4. This trend is consistent with the weighted F1 score and individual class performances, reinforcing that 0.4 is the most balanced threshold. Beyond the threshold of 0.6, accuracy drops more sharply, likely due to the model becoming overly conservative, leading to higher false negatives.

**Figure 11:**
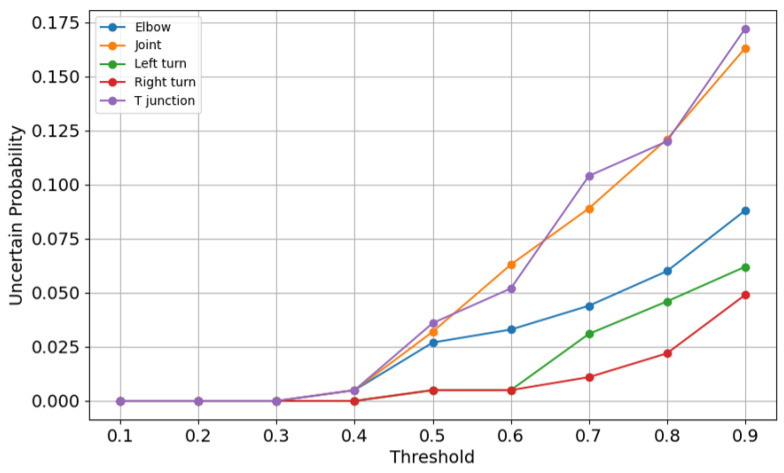
Uncertainty curves.

Figure 11 shows the uncertainty across a range of thresholds, revealing the model’s confidence levels across thresholds. At lower thresholds, the uncertainty probabilities are zero, indicating that the model is confident in its predictions. However, as thresholds increase, uncertainty grows sharply, particularly for joint, elbow, and T-junction. This finding suggests that higher thresholds introduce ambiguity, especially for categories with weaker separability. The rising uncertainty aligns with the declining F1 scores and accuracy observed at higher thresholds, further emphasizing the importance of selecting an optimal threshold.

In summary, the analysis reveals that the model performs exceptionally well across most categories, with the threshold of 0.4 offering the best balance between accuracy, F1 scores, and uncertainty. While elbow and right turn demonstrate consistent robustness, categories such as joint and T-junction present opportunities for further refinement to improve confidence and classification performance.

### 3.2. Smoothing Strategy Validation

**Figure 12:**
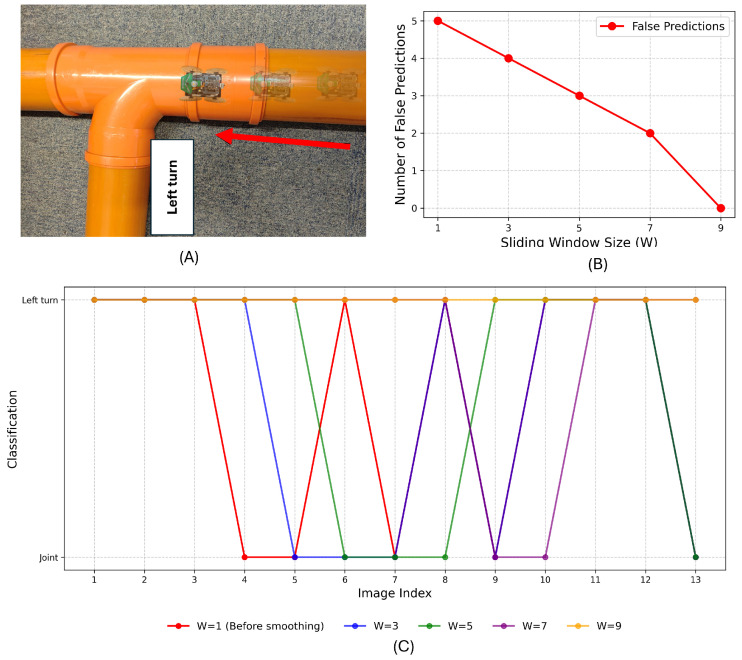
Smoothing method validation. (**A**) Experimental setup. (**B**) Number of false predictions. (**C**) The test result of sliding window smoothing method under different W values.

The model with a threshold of 0.4 was exported as an Arduino library and deployed on the Joey robot. To evaluate the performance of the sliding window smoothing method, the robot was tested in a pipe containing a left turn. The experimental pipe setup and the comparative outcome are depicted in Figure 12. The robot Joey moved in the direction of the red arrow. The detection results were recorded both before and after applying the smoothing technique. The figure demonstrates that the sliding window smoothing method effectively reduces misclassifications arising from individual frame predictions.

## 4. Discussion

The proposed TinyML-based method demonstrates significant progress in addressing the challenges of feature detection in small-diameter pipelines. By deploying a custom five-layer CNN model on a low-power ESP32 microcontroller, the method achieves a strong balance between accuracy and resource efficiency. The use of a sliding window smoothing strategy effectively minimizes the impact of transient misclassifications, ensuring reliable continue feature recognition even in challenging pipeline conditions such as low light.

While the proposed method demonstrates accurate and robust performance, further opportunities for optimization have been identified. For instance, the integration of depth information or range sensing could enhance the current vision-based approach by providing improved spatial perception and enabling precise localization within the pipeline. Such advancements represent promising avenues for future research, laying the groundwork for a more autonomous, reliable, and comprehensive framework for pipeline exploration using miniature robotic systems.

## 5. Conclusions

This paper presents a TinyML-based resource-efficient method for in-pipe feature detection, designed for miniature robots operating in small-diameter pipelines. The proposed method achieved a high detection accuracy of 97.1% by employing a custom five-layer CNN model, specifically optimized for deployment on resource-constrained hardware and successfully implemented on a miniature robot with an ESP32 microcontroller. A dedicated pipeline feature dataset was developed, encompassing diverse scenarios to support model training and evaluation. Furthermore, a comprehensive evaluation of CNN architectures identified a model that effectively balances computational efficiency and accuracy, with a peak RAM usage of 195.1 kB and a flash memory requirement of 427.9 kB, both of which are within the operational constraints of the ESP32 platform. To enhance method robustness, a sliding window smoothing strategy was integrated, mitigating transient misclassifications and ensuring stable performance under challenging conditions, such as low-light environments. These contributions underline the feasibility of deploying high-accuracy, low-resource machine learning models in constrained environments, providing a foundation for cost-effective and efficient solutions for autonomous pipeline exploration and inspection.

## Figures and Tables

**Table 1:** Comparison of CNN models.

Model	Accuracy	Loss	Inference Time (ms)	Peak RAM (kB)	Flash Usage (kB)
**Training from scratch**					
2 Conv	0.849	0.56	877	182.7	125.2
3 Conv	0.915	0.36	1144	184.7	99.7
3 Conv 160×160	0.933	0.52	4257	504.7	179.7
Conv+DW+PW+Conv	0.473	1.36	2742	184.9	66.3
3 Conv + SE	0.837	0.60	5606	295.5	118.2
3 Conv + ECA	0.864	0.53	1103	296.1	116.8
4 Conv	0.946	0.22	1232	188.3	135.2
5 Conv	0.971	0.20	1693	195.1	427.9
6 Conv	0.967	0.11	1586	208.3	1500.0
**Transfer learning**					
MobileNetV1 0.25	0.533	1.16	1083	124.7	306.3
MobileNetV1 0.1	0.323	1.55	744	52.5	105.9
MobileNetV1 0.2	0.263	1.73	744	52.5	105.9
MobileNetV2 0.05	0.584	2.60	1100	270.9	162.6
MobileNetV2 0.1	0.749	0.72	1135	280.8	212.6
MobileNetV2 0.35	0.900	0.39	1865	334.6	585.7

## Data Availability

The original contributions presented in the study are included in the article material. Further inquiries can be directed to the corresponding author.

## Abbreviations

The following abbreviations are used in this manuscript:

TinyML	Tiny Machine Learning
CNN	Convolutional Neural Network
CCTV	Closed-Circuit Television
ToF	Time-of-Flight
LVS	Laser Vision System
SURF	Speeded-Up Robust Features
SVM	Support Vector Machine
IoT	Internet of Things
Conv	Convolutional Layers
SE	Squeeze-and-Excitation
ECA	Efficient Channel Attention
DW	DepthwiseConv2D
PW	PointwiseConv2D
ROC	Receiver-Operating Characteristic
AUC	Area Under the Curve
TPR	True Positive Rate
FPR	False Positive Rate

## References

[1] Aitken J.M., Evans M.H., Worley R., Edwards S., Zhang R., Dodd T., Mihaylova L., Anderson S.R. (2021). Simultaneous localization and mapping for inspection robots in water and sewer pipe networks: A review. IEEE Access.

[2] Kazeminasab S., Sadeghi N., Janfaza V., Razavi M., Ziyadidegan S., Banks M.K. (2021). Localization, mapping, navigation, and inspection methods in in-pipe robots: A review. IEEE Access.

[3] Nguyen T., Blight A., Pickering A., Jackson-Mills G., Barber A.R., Boyle J.H., Richardson R., Dogar M., Cohen N. (2022). Autonomous control for miniaturized mobile robots in unknown pipe networks. Front. Robot. AI.

[4] Duran O., Althoefer K., Seneviratne L.D. Automated sewer pipe inspection through image processing. Proceedings of the 2002 IEEE International Conference on Robotics and Automation (Cat. No. 02CH37292).

[5] Tătar M., Pop A. (2016). Development of an in pipe inspection minirobot. Proceedings of the IOP Conference Series: Materials Science and Engineering.

[6] Kim S.H., Lee S.J., Kim S.W. (2018). Weaving laser vision system for navigation of mobile robots in pipeline structures. IEEE Sens. J..

[7] Ahrary A., Tian L., Kamata S.I., Ishikawa M. An autonomous sewer robots navigation based on stereo camera information. Proceedings of the 17th IEEE International Conference on Tools with Artificial Intelligence (ICTAI’05).

[8] Edwards S., Zhang R., Worley R., Mihaylova L., Aitken J., Anderson S.R. (2023). A robust method for approximate visual robot localization in feature-sparse sewer pipes. Front. Robot. AI.

[9] Li X.S., Nguyen T., Cohn A.G., Dogar M., Cohen N. (2023). Real-time robot topological localization and mapping with limited visual sampling in simulated buried pipe networks. Front. Robot. AI.

[10] Lee D.H., Moon H., Choi H.R. Landmark detection of in-pipe working robot using line-laser beam projection. Proceedings of the ICCAS 2010.

[11] Lee D.H., Moon H., Koo J.C., Choi H.R. (2013). Map building method for urban gas pipelines based on landmark detection. Int. J. Control Autom. Syst..

[12] Lee D.H., Moon H., Choi H.R. (2016). Landmark detection methods for in-pipe robot traveling in urban gas pipelines. Robotica.

[13] Chen F., Li S., Han J., Ren F., Yang Z. (2024). Review of lightweight deep convolutional neural networks. Arch. Comput. Methods Eng..

[14] Giordano M., Baumann N., Crabolu M., Fischer R., Bellusci G., Magno M. (2022). Design and performance evaluation of an ultralow-power smart IoT device with embedded TinyML for asset activity monitoring. IEEE Trans. Instrum. Meas..

[15] Lin J., Zhu L., Chen W.M., Wang W.C., Han S. (2023). Tiny machine learning: Progress and futures [feature]. IEEE Circuits Syst. Mag..

[16] Neuman S.M., Plancher B., Duisterhof B.P., Krishnan S., Banbury C., Mazumder M., Prakash S., Jabbour J., Faust A., de Croon G.C. Tiny robot learning: Challenges and directions for machine learning in resource-constrained robots. Proceedings of the 2022 IEEE 4th International Conference on Artificial Intelligence Circuits and Systems (AICAS).

[17] Pleterski J., Škulj G., Esnault C., Puc J., Podržaj P. (2023). Miniature Mobile Robot Detection using an Ultra-Low Resolution Time-of-Flight Sensor. IEEE Trans. Instrum. Meas..

[18] Avellaneda D., Mendez D., Fortino G. (2023). A TinyML deep learning approach for indoor tracking of assets. Sensors.

[19] Wang J., Chen C., Liu B., Wang J., Wang S. (2024). Pipeline Landmark Classification of Miniature Pipeline Robot *π*-II Based on Residual Network ResNet18. Machines.

[20] Howard A.G. (2017). Mobilenets: Efficient convolutional neural networks for mobile vision applications. arXiv.

[21] Sandler M., Howard A., Zhu M., Zhmoginov A., Chen L.C. Mobilenetv2: Inverted residuals and linear bottlenecks. Proceedings of the IEEE Conference on Computer Vision and Pattern Recognition.

[22] Qin D., Leichner C., Delakis M., Fornoni M., Luo S., Yang F., Wang W., Banbury C., Ye C., Akin B. MobileNetV4: Universal Models for the Mobile Ecosystem. Proceedings of the European Conference on Computer Vision.

[23] Pipebots (2022). Pipebots-Theme3. https://www.pipebots.ac.uk/.

[24] Hymel S., Banbury C., Situnayake D., Elium A., Ward C., Kelcey M., Baaijens M., Majchrzycki M., Plunkett J., Tischler D. (2022). Edge impulse: An mlops platform for tiny machine learning. arXiv.

[25] Goodfellow I., Bengio Y., Courville A. (2016). Deep Learning.

[26] Le M., Fokkens A. Neural models of selectional preferences for implicit semantic role labeling. Proceedings of the Eleventh International Conference on Language Resources and Evaluation (LREC 2018).

[27] Hu J., Shen L., Sun G. Squeeze-and-excitation networks. Proceedings of the IEEE Conference on Computer Vision and Pattern Recognition.

[28] Wang Q., Wu B., Zhu P., Li P., Zuo W., Hu Q. ECA-Net: Efficient channel attention for deep convolutional neural networks. Proceedings of the IEEE/CVF Conference on Computer Vision and Pattern Recognition.

[29] Chollet F. Xception: Deep learning with depthwise separable convolutions. Proceedings of the IEEE Conference on Computer Vision and Pattern Recognition.

